# SETIL: Italian multicentric epidemiological case–control study on risk factors for childhood leukaemia, non hodgkin lymphoma and neuroblastoma: study population and prevalence of risk factors in Italy

**DOI:** 10.1186/s13052-014-0103-5

**Published:** 2014-12-24

**Authors:** Corrado Magnani, Stefano Mattioli, Lucia Miligi, Alessandra Ranucci, Roberto Rondelli, Alberto Salvan, Luigi Bisanti, Giuseppe Masera, Carmelo Rizzari, Paola Zambon, Santina Cannizzaro, Lorenzo Gafà, Lia Lidia Luzzatto, Alessandra Benvenuti, Paola Michelozzi, Ursula Kirchmayer, Pierluigi Cocco, Pierfranco Biddau, Claudia Galassi, Egidio Celentano, Erni Guarino, Giorgio Assennato, Gigliola de Nichilo, Domenico Franco Merlo, Vittorio Bocchini, Franco Pannelli, Paola Mosciatti, Liliana Minelli, Manuela Chiavarini, Marina Cuttini, Veronica Casotto, Maria Valeria Torregrossa, Rosalia Maria Valenti, Francesco Forastiere, Riccardo Haupt, Susanna Lagorio, Serena Risica, Alessandro Polichetti

**Affiliations:** Medical Statistics & Cancer Epidemiology Unit - Department of Translational Medicine, CPO Piemonte and University of Eastern Piedmont, V. Solaroli 17, Novara, 28100 Italy; Section of Occupational Medicine, Department of Internal Medicine, Geriatrics and Nephrology, University of Bologna, Bologna, Italy; Occupational and Environmental Epidemiology Unit, ISPO Cancer Prevention and Research Institute, Florence, Italy; Cancer Epidemiology Unit, CPO Piemonte Novara, Novara, Italy; Paediatric Oncology-Haematology “Lalla Seràgnoli”, Policlinico S.Orsola-Malpighi, Bologna, Italy; Retired, formerly: IASI-CNR, Roma, Italy; Clinica Pediatrica, Università Milano Bicocca, Monza, Italy; A.O. San Gerardo, Fondazione MBBM, Monza, Italy; Epidemiologia, ASL di Milano, Milano, Italy; Registro Tumori del Veneto, Università di Padova, Padova, Italy; Lega Italiana per la Lotta contro i Tumori Onlus Sez, Provinciale di Ragusa, Ragusa Ibla, Italy; ASL 1 - Torino, Torino, Italy; Occupational and Environmental Epidemiology Unit, ISPO Cancer Prevention and Research Institute Firenze, Firenze, Italy; UOC Epidemiologia Ambientale, Dipartimento Epidemiologia Regione Lazio, Roma, Italy; Dipartimento Epidemiologia Regione Lazio, Roma, Italy; Dipartimento di Sanità Pubblica, Sezione di Medicina del Lavoro, Università di Cagliari, Cagliari, Italy; Servizio di Oncoematologia Pediatrica, Ospedale Microcitemico Cagliari, Cagliari, Italy; AOU S.Giovanni Battista e CPO Piemonte, Torino, Italy; S. O. Analisi e Monitoraggio, ARSAN - Agenzia Regionale Sanitaria della Campania, Napoli, Italy; Istituto Nazionale Tumori, Napoli, Italy; ARPA - Puglia, Bari, Italy; ASL BT. Dipartimento di Prevenzione, SPESAL, Barletta, Italy; Epidemiology, Biostatistics and Clinical Trials, IRCCS Azienda Ospedaliera Universitaria San Martino- IST Istituto Nazionale per la Ricerca sul Cancro, Genova, Italy; Epidemiology, Biostatistics and Clinical Trials, IRCCS AOU San Martino- IST Istituto Nazionale per la Ricerca sul Cancro, Genova, Italy; Registro Tumori di Macerata e Università di Camerino, Camerino, Italy; Università di Camerino, Dipartimento di Medicina Sperimentale e di Sanità Pubblica, Camerino, Italy; Dipartimento di Medicina Sperimentale - Sezione di Sanità Pubblica Università degli Studi di Perugia, Perugia, Italy; Dipartimento di Medicina Sperimentale - Sezione di Sanità Pubblica, Università degli Studi di Perugia, Perugia, Italy; Unità di Unità di Ricerca di Epidemiologia Perinatale, Ospedale Pediatrico Bambino Gesù, Roma, Italy; IRCCS Burlo Garofolo, Trieste, Italy; Dipartimento di Scienze per la Promozione della Salute Sez.Igiene, Università degli Studi di Palermo, Palermo, Italy; Dipartimento di Scienze per la Promozione della Salute Sez.Igiene, Università degli Studi di Palermo, Palermo, Italy; UOC Epidemiologia Ambientale, Dipartimento Epidemiologia Regione Lazio, Roma, Italy; Istituto Giannina Gaslini, Genova, Italy; National Centre for Epidemiology, Surveillance and Health Promotion, National Institute of Health, Rome, Italy; Retired, formerly: Department of Technology and Health, National Institute of Health, Rome, Italy

**Keywords:** Leukaemia, Non hogdkin lymphoma, Neuroblastoma, Epidemiology, Risk factors

## Abstract

**Background:**

Aetiology of childhood leukaemia and childhood neoplasm is poorly understood. Information on the prevalence of risk factors in the childhood population is limited. SETIL is a population based case–control study on childhood leukaemia, conducted with two companion studies on non-Hodgkin Lymphoma (NHL) and neuroblastoma. The study relies on questionnaire interviews and 50 Hz magnetic field (ELF-MF) indoor measurements. This paper discusses the SETIL study design and includes descriptive information.

**Methods:**

The study was carried out in 14 Italian regions (78.3% of Italian population aged 0–10). It included leukaemia, NHL and neuroblastoma cases incident in 0–10 year olds in 1998–2001, registered by the Italian Association of Paediatric Haematology and Oncology (AIEOP) (accrual over 95% of estimated incidence). Two controls for each leukaemia case were randomly sampled from the Local Health Authorities rolls, matched by gender, birthdate and residence. The same controls were used in NHL and neuroblastoma studies. Parents were interviewed at home on: physical agents (ELF-MF and ionizing radiation), chemicals (smoking, solvents, traffic, insecticides), occupation, medical and personal history of children and parents, infectious diseases, immunizations and associated factors. Occupational exposure was collected using job specific modules. ELF-MF was measured in the main rooms (spot measurement) and close to child’s bed (48 hours measurement).

**Results:**

The study included: 683 leukaemia cases (87% ALL, 13% AnLL), 97 NHL, 155 neuroblastomas, and 1044 controls.

ELF-MF long term measurements were obtained for 61.1% of controls and 81.6% of leukaemia cases; 8.8% of controls were exposed at over 0.1 microTesla (μT), 3.5% and 2.1% at respectively over 0.2 and 0.3 μT. 25% of controls’ fathers had smoked over 10 cigarettes/day during the year of conception, varying according to education and region. Maternal smoking was less common (71.4% did not smoke in pregnancy). Maternal passive smoking during pregnancy was reported by 31.2% of controls; the child’s passive smoking for 28.6%.

Occupational exposure to solvents was estimated in 18.3% of controls’ fathers and 7.7% of mothers. Contact with public was more frequent among mothers (36.1%) than fathers (23.4%).

**Conclusions:**

SETIL represents a data source on exposure of Italian children to a broad array of potential carcinogenic factors.

**Electronic supplementary material:**

The online version of this article (doi:10.1186/s13052-014-0103-5) contains supplementary material, which is available to authorized users.

## Background

Despite intensive research, the aetiology of childhood neoplasms is still poorly known, even in the case of frequent tumour types such as leukaemia, non-Hodgkin Lymphomas (NHL) [1], and neuroblastoma [2]. Several possible risk factors in the aetiology of childhood leukaemia, have been investigated including ionizing radiations in pregnancy and after birth, Extremely Low-Frequency magnetic fields (ELF-MF) and radiofrequency fields, solvents, pesticides and other chemicals in the environment, infectious agents, immunizations and related factors. With the notable exception of ionizing radiation, research results were not conclusive [1,3-5]. Strong support for an association between exposure to common infections in early childhood (as estimated by day-care attendance) and a reduced risk of acute lymphoblastic leukaemia was provided by a recent meta-analysis of 14 case–control studies [6]. The aetiology of childhood NHL is also poorly known, with the notable exception of Burkitt lymphoma and EBV [3]. Epidemiological studies on neuroblastoma also investigated a wide range of putative risk factors, including: socioeconomic conditions, reproductive history, diagnostic x-rays, maternal exposures in pregnancy, child’s characteristics, parental occupation and related exposures, all with inconsistent results; a protective effect was observed from breastfeeding and folates in pregnancy [2].

From a population perspective, the prevalence of exposed children is as important as the estimation of risk since it is the basis for the correct estimation of population attributable fraction and for preventive measures under the framework of the precautionary principle.

A multicentre, population-based case–control epidemiological study (SETIL - Studio Epidemiologico sui Tumori Infantili Linfoemopoietici) was carried out in Italy to investigate risk factors for selected childhood neoplasms. The main study focused on leukaemia and was accompanied by two smaller studies on NHL and neuroblastoma. The study relied on questionnaire based interviews to parents of the study subjects and indoor measurements of 50 Hz ELF-MF.

Two methodological side studies, including subsets of the main study, were performed on benzene [7] and on gamma radiation exposure [8], in order to obtain information on the occurrence of these exposures and their correlation with ELF-MF.

The present paper illustrates the design of the SETIL study, provides descriptive information on subjects included in the main case–control study, and presents the prevalence of exposure of controls to some of the investigated factors.

## Methods

### Cases and controls

SETIL is a population based case–control study that was carried out in 14 Italian regions (Piedmont, Liguria, Lombardy, The Venetian Region, Friuli Venezia Giulia, Emilia Romagna, Tuscany, Umbria, The Marches, The Latium, Campania, Apulia, Sicily and Sardinia). Cases of leukaemia, non-Hodgkin Lymphoma (NHL) and neuroblastoma in children aged 0–10 newly diagnosed between August 1998 and December 2001 (with minor regional differences) were eligible for recruitment. The network of paediatric oncology centres affiliated to the Italian Association of Paediatric Haematology and Oncology (AIEOP), i.e. the national network of the Italian childhood cancer centres, was the source of cases. AIEOP runs a nation wide database which records all the new cases of paediatric cancer diagnosed or treated in the network. Cases of paediatric neoplasm are recorded on admission at any AIEOP unit using a common registration form which includes the patient’s personal identification data, as well as diagnosis and treatment information. Each new patient is registered using electronic case report forms in the AIEOP “Mod.1.01 Registry” web-based database hosted on the AIEOP website (www.aieop.org) [9]. Estimated coverage of the AIEOP registry in the period of the SETIL study was 94% for leukaemia, 98% for neuroblastoma and 100% for NHL [10]. The list of children eligible for the study was provided monthly by the AIEOP database at each Regional Research Unit by means of authorized access to the report area of the database. Only in the Lazio Region a supplementary *ad hoc* search of eligible cases was carried out through the hospital discharge files of the main Rome paediatric hospital (not in the AIEOP network at the time).

Controls were chosen at random from the population residing in each region, using the Local Health Authority (LHA) rolls. Two controls were randomly sampled for each leukaemia case, matched by gender, date of birth (±15 days) and LHA of residence. The resulting set of controls was used as a pool in the studies on NHL and neuroblastoma, controlling analyses for age, gender and area of residence. Non participating controls were not substituted.

Date of diagnosis was defined according to the clinical information provided by AIEOP database with cases; for controls a “pseudo diagnosis date” was set equal to the date of diagnosis of the corresponding matched case. Controls retained the assigned pseudo-diagnosis date for the analyses for lymphoma and neuroblastoma substudies. Information was collected until child’s diagnosis (or “pseudo diagnosis date” for matched controls). A “reference date” was used to align the time of interest when describing child’s typical day and use of (or exposure to) electrical appliances and other exposures. It was defined as one year before diagnosis (if case) or “pseudo diagnosis date” (if controls). For children under 2 at diagnosis, the reference date was corresponding to the age of diagnosis divided by two.

### Study presentation to index families

Regarding the cases, the study was explained to child’s parents by the attending oncologist, usually after the induction phase of treatment. Child’s parents were contacted by the study staff only after the approval of the attending oncologist. Families of children dying before the study presentation were not excluded from the study but the study presentation was delayed at discretion of the attending clinician until the parents’ psychological conditions allowed for it.

Controls’ general practitioners (GP) were informed by mail about the child’s enrolment in the study and were asked to report their objections, if any. In such instances the study local coordinator contacted the GP in order to decide on the child’s inclusion; confirmed GP’s refusal was accepted. GPs were welcome to inform the families about the study but this was not required.

### Interview

After medical approval, a research assistant mailed a letter to child’s parents with detailed information on the study and, a few days later, called them in order to verify consent and arrange a suitable time for the interview. Interviewers were instructed to carry on several (at least 5) telephone call attempts when needed, at different times and on different days before declaring the subject as non responder. Interviews always took place in the home. Interviewers were not blind about child’s status as blinding is practically infeasible in similar studies on childhood neoplasms. They were instructed (see later) in order to avoid any differences when contacting case or control families. A consensus form was signed before the interview.

Although we wished to interview both parents at the same time, we soon realized that this was not always possible. When appropriate, further information regarding the non participating parent was provided by the attending spouse and confirmed or elicited over the phone.

The interviews were conducted according to a standardized questionnaire and were carried out by specifically trained interviewers. The questionnaire was designed for a duration of between 1 hour and 1 hour and 30 minutes.

Items included were:

- Personal and medical history, including reproductive history, pregnancy duration, child conditions at birth, breast feeding, X-rays, childhood diseases and immunizations;
- Exposure to chemical substances at home and in the environment, with focus on solvents, pesticides, traffic pollution, environmental tobacco smoke;
- Checklist on maternal (during pregnancy) and child’s home exposure to electrical appliances;
- School attendance, including age at first attendance, school and class size;
- Parental lifelong occupational history, with detailed job and workplace description;
- Parental educational level, measured as the highest level of education attained;
- Child’s lifelong and maternal (during pregnancy) residential history, with details on all dwellings, including full addresses, location (urban or rural), indicators of traffic pollution, pesticide environmental exposure and proximity to power lines and broadcasting stations.

Information on parental occupational exposure was collected using different tools: first a lifelong list of occupations (job title and industrial activity) was gathered with corresponding dates of commencement and finish. For each occupation dating from one year before conception until child’s diagnosis, further details were collected using job specific modules focused on exposure to solvents, pesticides and other chemicals. Questions on potential ionizing radiation exposure and on EMF sources were also included. Regarding agricultural work, crop-specific modules were prepared, focussing on pesticides and insecticides. The questionnaire is available on request.

### ELF magnetic field measurement

ELF-MF was measured by the interviewer in the main rooms of the house (usually chosen among the living room, parents’ and child’s bedroom and kitchen), according to a defined protocol including measurements taken in the centre and corners of each room, with lights and electrical appliances on and off (spot measurements). An ELF-MF meter was left under or close to child’s bed for a 48 hours measurement (long-time measurements). Two types of portable meters were used: EMDEX II® for spot measurements and either EMDEX-Lite® or EMDEX II® for long term measurements. Both meters take an instantaneous field measurement on three orthogonal axes at fixed intervals. Sampling interval was set to 4 seconds for spot and to 30 seconds for long term measurements. In the statistical analyses measurements were summarized according to different metrics (arithmetic and geometric means and percentiles). Instantaneous measurements below the detection limit (0.01 μT) to compute geometric means were substituted with the value of 0.0001 μT. All meters were calibrated in laboratory every 6 months and whenever unexpected results were observed. All sets of measurements were inspected graphically using the EMCALC© software (v.95 and later): defective sets of measurements were excluded and, if possible, repeated. If repetition was not feasible, the measurement was truncated. Evaluation was performed blindly in a laboratory setting by a trained physicist. The protocol for ELF-MF measurement was defined during a pilot study including a random sample of over 100 subjects in 5 regions [11].

The questionnaire included questions on maternal use of mobile and cordless phones during pregnancy and on the distance of dwellings from radio towers or mobile phone stations.

### Expert assessment of parental occupational exposure

Occupational exposure to a list of exposures of apriori interest was estimated with expert assessment of the information in the questionnaire and in the job specific modules. Assessment was performed by a team of industrial hygienists and was conducted blindly as to the case or control status. The same procedure was used for both parents.

### Interviewer training

Interviewers were trained during a residential course that took place before the study commencement and was repeated yearly. The first course edition focused on the general principles of the epidemiological study, on the psychological approach to cases’ and controls’ families, on the questionnaire and on the use of ELF-MF meters. Subsequent editions were more focused on the discussion and the analysis of practical work, based on interviewers’ experience.

### Power and data analysis

The power of the study was estimated on the basis of the number of cases expected in the study period (696 ALL, 113 AnLL, 78 NHL and 219 neuroblastoma) [9,10]. Regarding leukaemia, the sample size was large enough to detect moderately increased relative risks as statistically significant (e.g. RR > =2.8 with 1% of exposed and 1.7 with 5%; α=.05, 1-β=.80).

Study management and data registration were carried out using Microsoft Access; statistical analyses using EMCALC® (V.95 and later) (for ELF-MF measurements data), SAS 9.2 and Stata 10.

## Ethical committee

The SETIL study was authorized by the Ethical Review Board for the Piedmont Region (authorization n. 2886, on 15/2/1999; letter n. 1852/28.3 on 17/2/1999) and later by the corresponding board of each participating research unit.

## Results

The study included 14 of the 20 Italians regions, corresponding to 78.3% of the total Italian population in the 0–10 age range (86.9% in Northern Italy, 100% in Central Italy and 60.8% in Southern Italy). In some regions the study was limited to limited areas or was conducted for less than the three year period anticipated according to the protocol Table 1. Lombardy weighed most in the study, followed by the Venetian Region (Veneto). For logistic reasons two research units were active in Sicily, respectively for the Province of Palermo and for the eastern part of the region.

**Table 1:** **SETIL study: cases and controls eligible in the study and distribution of non participating subjects by reason of non participation**

	**Controls**		**Leukaemia**		**NHL**		**Neuroblastoma**	
	**n**	**%**	**n**	**%**	**n**	**%**	**n**	**%**
Total eligible	1,475	-	745	-	116	-	207	-
Participant	1,044	70.8%	683	91.7%	97	83.6%	155	74.9%
Non participant	431	29.2%	63	8.3%	19	16.4%	52	25.1%
Reason for non participation								
Family refusal	303	70.3%	26	41.3%	6	31.6%	21	40.4%
Medical refusal	27	6.2%	7	11.1%	3	15.8%	6	11.1%
Dying child, medical consensus not obtained	0	0.0%	21	33.3%	4	21.1%	11	21.1%
Adopted, no information on natural family	4	0.9%	2	3.2%	1	5.3%	0	0.0%
Untraced or not approached	94	21.8%	6	9.5%	1	5.3%	8	15.4%
Other and unknown	3	0.7%	0	0.0%	4	21.1%	6	11.5%

In the study period the eligible cases were as follows: 745 children affected by leukaemia, 116 by NHL and 207 by neuroblastoma. One thousand four hundred seventy five controls were randomly selected and individually matched to leukaemia cases. These numbers excluded the subjects found non eligible during the study process. Participation was 91.7% for leukaemia, 83.6% for NHL and 74.9% for neuroblastoma cases, and 70.8% for controls. Non participants were not substituted. Family refusal was the most common reason of non-participation among controls (70.3% of non participants) while it was less frequent among non participating cases (leukaemia: 41.9%, NHL: 31.6%, neuroblastoma: 40.4%). Denied medical approval, was obviously more frequent among non-participating cases (leukaemia 45.2%; NHL 36.9%; neuroblastoma 33.7%), than among controls (6.3%). (Table 1). The relative distribution of participant cases and controls by region is presented in the additional material (see Additional file 1: Table S1). All large Italian geographical areas (North, Centre, South and the Islands) were included but the coverage was more comprehensive for the Northern and Central ones.

Due to the eligible age-range (0–10 years), the great majority of 683 leukaemia cases participant in the study were of the Acute Lymphoblastic Leukaemia (ALL) subtype (594 cases, 87%), with 7 cases of Acute Hybrid Leukaemia (included in the AnLL group for simplicity), and 82 cases (13%) of Acute non Lymphoblastic Leukaemia (AnLL). Frequency distribution of AnLL by cytological subtype was: M1: 7.3%, M1-M2: 14.6%, M2: 15.9%, M3: 19.5%, M4: 12.2%, M5: 17.1%, M6: 1.2%, M7: 2.4, other and unclassified: 9.8%.

Table 2 presents the distribution of participating subjects by selected characteristics. The attained educational level was higher among controls than cases. The level of education among controls was: primary education for 44.3% of fathers and 38.3% of mothers; secondary for 40.6% and 48.2% and tertiary for 14.5% and 13.3%. The time lag between diagnosis or pseudo diagnosis and interview was shorter for cases, especially for leukaemia, than for controls: cumulative proportions of interview at 18 months from diagnosis were 75.8% for leukaemia, 54.9% for controls, 55.7% for NHL and 55.5% for neuroblastoma cases.

**Table 2:** **SETIL study: frequency distribution of participating subjects by condition, gender, child’s age, year of diagnosis, lag between diagnosis and interview (months), parental education level, maternal age at child’s birth**

		**Controls**		**Leukaemia**		**NHL**		**Neuroblastoma**	
		**n**	**%**	**n**	**%**	**n**	**%**	**n**	**%**
Gender	Girls	482	46.2	313	45.8	19	19.6	63	40.6
	Boys	562	53.8	370	54.2	78	80.4	92	59.4
	Total	1044	100.0	683	100.0	97	100.0	155	100.0
Age	[0; 2)	156	14.9	95	13.9	5	5.2	88	56.8
	[2; 4)	351	33.6	243	35.6	10	10.3	33	21.3
	[4; 6)	233	22.3	146	21.4	14	14.4	25	16.1
	[6; 11)	304	29.1	199	29.1	68	70.1	9	5.8
Year of diagnosis	1998	109	10.4	67	9.8	7	7.2	20	12.9
	1999	314	30.1	212	31.0	32	33.0	35	22.6
	2000	342	32.8	229	33.5	32	33.0	57	36.8
	2001	279	26.7	175	25.6	26	26.8	43	27.7
Lag diagnosis - interview (months)	[0; 6)	30	2.9	29	4.2	5	5.2	5	3.2
	[6; 12)	170	16.3	192	28.1	21	21.6	30	19.4
	[12; 18)	373	35.7	297	43.5	28	28.9	51	32.9
	[18 and over]	471	45.1	165	24.2	43	44.3	69	44.5
Maternal education	Primary	400	38.3	320	46.9	43	44.3	66	42.6
	High school	503	48.2	285	41.7	44	45.4	75	48.4
	University d.	139	13.3	78	11.4	10	10.3	14	9.0
	Missing data	0	0.0	0	0.0	0	0.0	0	0.0
Paternal education	Primary	463	44.3	340	49.8	47	48.5	81	52.3
	High school	424	40.6	268	39.2	39	40.2	57	36.8
	University d.	151	14.5	70	10.2	11	11.3	16	10.3
	Missing data	6	0.6	5	0.7	0	0.0	1	0.6
Maternal age at child’s birth	Mean (SD)	29.66	(4.76)	29.84	(4.91)	29.84	(4.86)	30.10	(4.75)
Paternal age at child’s birth	Mean (SD)	33.95	(5.40)	33.13	(5.61)	32.66	(5.27)	33.79	(5.52)

Parents’ participation was recorded separately for each section of the interview. Overall, mothers attended the interview more often, with participation in the range 98.2% of interviews (section on dwellings) to 99.0% (maternal occupation), while fathers’ participation was in the range 62.5% (section on dwellings) to 69.5% (section on general information). Additional information on occupation was provided at a later time by 4% of fathers. No differences were observed for maternal participation by case/control status while limited differences were observed for paternal participation, that was lower for controls and also for lymphoma cases (Additional file 1: Table S2).

The participation to ELF-MF long term measurements is summarized in the supplementary material (Additional file 1: Tables S3). The overall proportion of eligible subjects with measurement is lower for controls (61%) and highest for the leukaemia cases (82%), depending on the participation in the interview: among those who accepted it, the proportion without measurements is similar in the cases and controls.

Prevalence of infectious diseases until reference time are summarized in Table 3. Chickenpox was reported for 35.0% of controls, followed by mumps (11.4%). For all diseases included in the checklist, in most instances the diagnosis was reported as “confirmed by a medical doctor”. High temperature and illness affecting the upper airways (colds, throat infections, sinusitis, ear infections…) in the first year of life were reported by 62.5% of controls, with medical confirmation in 93.1%. More serious diseases of likely infectious origin, such as bronchitis (16%) or pneumonia (0.8%) were much rarer.

**Table 3:** **SETIL study: prevalence of common infections among controls**

		**Yes**		**Missing**		**MD confirmation**	
		**N**	**%**	**N**	**%**	**N**	**%**
At any time before reference date	Measles	62	5.9	5	0.5	61	98.4
	Pertussis	67	6.4	4	0.4	65	97.0
	Rubella	49	4.7	6	0.6	44	89.8
	Chicken pox	365	35.0	1	0.1	355	97.3
	Mumps	119	11.4	5	0.5	113	95.0
	Scarlet fever	80	7.7	6	0.6	78	97.5
	Mononucleosis	12	1.2	3	0.3	11	91.7
	Herpes Labialis	25	2.4	1	0.1	15	60.0
	Herpes Oral mucosae	44	4.2	2	0.2	38	86.4
First year of life.	Febrile diseases - respiratory tract	653	62.6	9	0.9	608	93.1
	Bronchitis	167	16.0	5	0.5	165	98.8
	Pneumonia	8	0.8	6	0.6	8	100.0
	G.U. infections	43	4.1	6	0.6	42	97.7
	Febrile diseases - unspecified	46	4.4	9	0.9	36	78.3

Percentages are computed over the number of participant controls.

Parental smoking habits were considered focussing on the year of conception for the father and on the pregnancy for the mother (Table 4): two different indicators were used to summarize questionnaire information: for fathers cigarettes/day during the year of conception and for mothers the cumulative number of packs summed over the pregnancy. Over a quarter of controls’ fathers reported smoking over 10 cigarettes/day during the year of conception: the proportion varied according to education (a higher smoking prevalence was observed among the less educated) and region (North to South increasing gradient). Maternal smoking was less common (71.4% non smokers, 25% in the category up to 100 packs and 3.2% in the category over 100 packs as cumulative consumption during pregnancy) and their level of education appeared to have a smaller impact (higher smoking prevalence among the less educated) and region (higher smoking prevalence in Central Italy regions).

**Table 4:** **SETIL study parental smoking status of controls classified according to child’s residence, and parental educational level**

		**Paternal smoking**							**Maternal smoking**							
		**Nonsmoker** ^**a**^		**Moderate smoker** ^**b**^		**Heavy smoker** ^**c**^		**Missing**	**Nonsmoker** ^**d**^		**Moderate smoker** ^**e**^		**Heavy smoker** ^**f**^		**Missing**	**Total**
		**N**	**%**	**N**	**%**	**N**	**%**	**N**	**N**	**%**	**N**	**%**	**N**	**%**	**N**	**N**
Total		571	55.3	188	18.2	273	26.5	12	745	71.4	265	25.4	33	3.2	1	1044
Education	Less than high school	211	45.8	80	17.3	170	36.9	2	275	67.9	115	28.4	15	3.7	0	405
	High school	253	60.2	80	19.1	87	20.7	4	372	74.4	114	22.8	14	2.8	1	501
	University	107	71.3	27	18.0	16	10.7	1	96	70.6	36	26.5	4	2.9	0	136
	Missing	0		1		0		5	2		0		0		0	2
Residence	North. Italy (excl. Lombardy)	155	62.5	40	16.1	53	21.4	2	186	74.4	56	22.4	8	3.2		250
	Lombardy	146	57.3	50	19.6	59	23.1	5	190	73.4	60	23.2	9	3.5	1	260
	Central Italy	140	55.3	47	18.6	66	26.1	4	171	66.5	82	31.9	4	1.6		257
	Southern Italy	130	47.1	51	18.5	95	34.4	1	198	71.5	67	24.2	12	4.3		277

Paternal smoking is referred at child conception and maternal smoking during pregnancy.

Percentages are calculated by row, on subjects with available information.

^a^Nonsmoker category includes never smokers and fathers who quitted smoking at least the year before conception.

^b^Moderate smoker category includes fathers who quitted smoking in the year of the conception and smokers of 1–10 cigarettes per day in the period of the conception.

^c^Heavy smoker category includes fathers that smoked 11 or more cigarettes per day in the period of the conception.

^d^Nonsmoker category includes never smokers and mothers who quitted smoking at least the year before the pregnancy.

^e^Moderate smoker category includes mothers who quitted smoking in the year of the pregnancy and women that smoked 1–100 packets of cigarettes during the pregnancy.

^f^Heavy smoker category includes mothers that smoked 101 or more packets of cigarettes during the pregnancy.

Passive smoking for pregnant mothers was reported by 31.2% of controls, with 9.4% in the highest category of 4 hours of exposure per day (Table 5). Child’s passive smoking was summarized in a cumulative index counting a number of cigarettes up to the reference date, considering all sources (most notably parents and relatives). Exposure to passive smoking was reported for 28.6% of controls, with 11.4% in the highest category of over 7000 cigarettes (Table 5). For both maternal and child’s passive smoking the trends were as for active parental smoking: higher exposure among the less educated and North to South increasing gradient.

**Table 5:** **SETIL study prevalence of exposure to passive smoking among controls: maternal exposure during pregnancy and child’s exposure to environmental tobacco smoke, by child’s age, residence, and parental educational level**

		**Maternal exposure to environmental tobacco smoke during pregnancy**									
		**Not exposed** ^**a**^		**<2.00 hours/day** ^**a**^		**2.00-3.99 hours/day** ^**a**^		≥**4.00 hours/day** ^**a**^		**Missing**	**TOTAL**
		**N**	**%**	**N**	**%**	**N**	**%**	**N**	**%**	**N**	**N**
Total		710	68.8	122	11.8	103	10.0	97	9.4	12	1044
Education (Mother)	Less than high school	249	62.4	48	12.0	55	13.8	47	11.8	6	405
	High school	349	70.4	66	13.3	35	7.1	46	9.3	5	501
	University	110	81.5	8	5.9	13	9.6	4	3.0	1	136
	Missing	2		0		0		0		0	2
Residence	Northern Italy (excl. Lombardy)	180	72.6	28	11.3	18	7.3	22	8.9	2	250
	Lombardy	199	77.1	20	7.8	18	7.0	21	8.1	2	260
	Central Italy	179	70.7	22	8.7	26	10.3	26	10.3	4	257
	Southern Italy	152	55.7	52	19.0	41	15.0	28	10.3	4	277
		**Exposure of children to environmental tobacco smoke (as total number of cigarettes)**									
		**Not exposed** ^**a**^		**<1000 cigarettes** ^**a**^		**1000-6999 cigarettes** ^**a**^		≥**7000 cigarettes** ^**a**^		**Missing**	**Total**
		**N**	**%**	**N**	**%**	**N**	**%**	**N**	**%**	**N**	**N**
Total		740	71.4	91	8.8	88	8.5	118	11.4	7	1044
Age	[0–2)	119	76.8	19	12.3	15	9.7	2	1.3	1	156
	[2–4)	255	73.1	41	11.7	25	7.2	28	8.0	2	351
	[4–6)	161	69.1	24	10.3	13	5.6	35	15.0	0	233
	[6–11)	205	68.3	7	2.3	35	11.7	53	17.7	4	304
Education (Both parents)	Both parents less than high school degree	283	61.1	52	11.2	51	11.0	77	16.6	3	466
	At least one high school degree	337	79.5	29	6.8	25	5.9	33	7.8	3	427
	At least one university degree	120	80.0	10	6.7	12	8.0	8	5.3	1	151
	Missing	0		0		0		0			
Residence	Northern Italy (excl. Lombardy)	186	75.0	20	8.1	18	7.3	24	9.7	2	250
	Lombardy	211	81.1	22	8.5	11	4.2	16	6.1	0	260
	Central Italy	197	77.6	10	3.9	21	8.3	26	10.2	3	257
	Southern Italy	146	53.1	39	14.2	38	13.8	52	18.9	2	277

Percentages are calculated by row. Information on maternal exposure missing for 15 controls.

^a^: Categories of exposure to Environmental Tobacco smoke are based on tertiles of exposure among exposed controls, rounded to the nearest half hour (maternal exposure) or to the nearest thousand (child’s exposure).

Table 6 presents exposure ELF-MF, according to the results of long term ELF-MF measurements. Results were summarized according to the following metrics: arithmetic and geometric mean, median and 90° and 95° percentile, grouped in four classes (<0.1, 0.1-0.2, 0.2-0.3 and over 0.3 μT). Data from the arithmetic mean showed 8.8% of controls were exposed at over 0.1 μT, 3.5% at over 0.2 μT and 2.1% at over 0.3 μT.

**Table 6:** **SETIL study prevalence of exposure to ELF-MF among controls (Total n.: 904 with ELF-MF measurements)**

	**Results of ELF-MF measurements (μT)**							
	**<=0.1**		**(0.1 - 0.2]**		**(0.2 - 0.3]**		**>0.3**	
**Metric**	**N**	**%**	**N**	**%**	**N**	**%**	**N**	**%**
Arithmetic mean	824	91.15	48	5.31	13	1.44	19	2.10
Geometric mean	837	92.58	43	4.76	11	1.22	13	1.44
Median	842	93.14	36	3.98	11	1.22	15	1.66
90° percentile	754	83.41	91	10.07	19	2.10	40	4.42
95° percentile	721	79.76	112	12.39	27	2.99	44	4.86
99° percentile	659	72.90	126	13.94	53	5.86	66	7.30

The distribution of controls’ parents according to job description and condition from one year before conception to the child’s diagnosis is presented in the supplementary material (Additional file 1: Table S4 and S5, respectively). Almost all fathers of control children were employed at the study time: only 1.3% described themselves as “never employed”. On the contrary 13.3% of mothers reported themselves as “never employed”, with large regional variations.

Occupational exposure to solvents (at any time from one year before conception until child’s diagnosis) was observed for 18.3% of controls’ fathers and 7.7% of controls’ mothers (Table 7). Corresponding proportions of exposed to benzene were 3.3% and 0.3%. Contact with public was more frequent among mothers (36.1%) than fathers (23.4%).

**Table 7:** **SETIL study prevalence of occupational exposure of among control’s parents**

**EXPOSURE**	**FATHER**		**MOTHER**	
	**N**	**%**	**N**	**%**
Any solvent	189	18.3	80	7.7
- Aromatic hydrocarbons	98	9.5	18	1.7
- Chlorinated hydrocarbons	61	5.6	33	3.2
- Technical hydrocarbons	92	8.9	17	1.6
- Aliphatic hydrocarbons	69	6.7	13	1.2
- Derivate oxygenate hydrocarbons	75	7.2	45	4.3
Benzene	34	3.3	3	0.3
Chloroform	2	0.2	0	0
Dichloromethane	14	1.3	15	1.4
Dioxane	1	0.1	0	0
Styrene	9	0.9	3	0.3
Tetracloroethane	25	2.4	5	0.5
Trichloroethylene	27	2.6	8	0.8
1,1,1 trichloroethane	15	1.4	1	0.1
Toluene	73	7.0	12	1.1
Xylene	69	6.6	10	1.0
Acetamide	0	0	0	0
Acrylonitrile	7	0.7	0	0
P. aminoazobenzene	0	0	1	0.1

Percentages are computed over the number of participant controls.

## Discussion

This paper presents methods and summarizes descriptive results of the SETIL Italian case–control study on childhood leukaemia, NHL and neuroblastoma. It also provides a first description of the extent of exposure of Italian children in pre-school and primary school age to some putative risk factors for leukaemia.

Distribution of cases by age and gender was very close to expectations according to the population cancer registry data [12].

Participation in the study was almost complete for leukaemia cases and over 80% for lymphoma and neuroblastoma cases. The difference is likely to depend on the longer time requested for the therapy and the larger proportion of children that were not in remission. Attending clinicians recommended not to contact cases’ families until the conclusion of the induction phase of the therapy.

In our study controls were sampled from the LHA rosters and therefore participation rate could be accurately measured. Case names were selected in the same data base used for sampling controls, to check the correspondence between case and control sources, and were always found. Participation of controls’ families reached 70.8%, a figure that is similar or higher than the results observed in other studies on the topic with population based recruitment. Linet et al. [13] obtained an interview from 72.8% of controls and a ELF-MF measurement from 63%. In the UK study [14] participation of controls was 64%, while it was 62.0% in the German study by Schuz et al. [15]. Ma et al. observed in California that control participation was 78.2% of those who accepted a first contact with the study [16], corresponding to an estimated fraction of 49% of those eligible.

Participant control parents’ education was comparable to figures provided by the Italian National Institute of Statistics (ISTAT) for the general population of a corresponding age. In the 2001 census, the proportion of subjects with high school diploma or university degree in age class 19–34 was 50.1% for men and 57.9% for women [17]. With reference to 2004–2005 period figures were also available for University and High school degree, separately. The proportion of men with University degrees or Tertiary Education was: 2.8% in the 20–24 age group, 11.6% in the 25–25 age group, 13.5%, in the 30–34 age group, and 12.2%, in the 35–39 age group. Corresponding proportions with high school diploma were: 67.5% in the 20–24 age group, 43.8% in the 25–29 age group, 37.8% in the 30–34 age group and 34.0% in the 35–39 age group. In women the proportion with a University Degree was: age 20–24: 3.9%, age 25–29: 14.2%, age 30–34: 17.2%, age 35–39: 12.3%; corresponding proportions with high school diploma were: age 20–24: 69.0%, age 25–29: 50.9%, age 30–34: 40.5%, age 35–39: 34.1%. [18].

The database of ELF-MF measurements included 904 controls, corresponding to an overall participation of 61.1%. Almost 90% of the interviewed subjects also participated in the ELF-MF measurements. To our knowledge, this is the largest Italian database on long term measurements of ELF-MF exposure in the general population and will be valuable for additional analyses on ELF-MF exposure by region and by other covariates, such as socioeconomic variables. The proportion of controls with estimated exposure to ELF-MF over 0.3 μT was 1.44% (Geometric mean; 95% CI 0.84% - 2.45%) in the present study, higher than, but not incompatible with the proportion of controls living in dwellings with long term measured ELF magnetic fields above 0.3 μT observed in the European studies included in the pooled analyses by Greenland et al. [19], Ahlbom et al. [20], and Kheifets et al. [21]. The difference is sensitive to the metric used to summarize the individual measures, therefore we decided to present results according to different metrics: in the studies included in the Greenland et al. [19] pooled analysis, the proportion of controls living in dwellings with ELF-MF higher than 0.3 μT (summarized using the arithmetic mean) was estimated in 1.33%, while in the present study the corresponding proportion was 2.10% (95% CI: 1.35% - 3.26%). If all studies with measured fields in those pooled analyses are considered, the proportion of controls exposed to over 0.3 μT increased to 1.9% (our computation from tabulated data), because of the larger proportions of controls in the right tail of the exposure distribution recorded in non-European studies.

In our study we focused on paternal smoking at the conception and on maternal smoking during pregnancy: 55.3% of controls fathers and 71.4% of mothers stated at the interview they were non smokers while 44.7% and 28.5% described themselves as smokers. These figures are close to reports from Italian surveys on corresponding periods. Sardu et al. [22] estimated the proportion of smokers by age group, gender and period, based on a national survey from ISTAT: in 1995 (centre of the year-of-birth distribution in our study) prevalence of smokers in men was 43% in age 20–24, 47% in age 25–29 and 45% in age to 30–35. Corresponding figures in women were 24%, 30% and 32%, close to our results. A trend was observed in education levels, more marked in men but also present in women. In the 2000 survey on smoking habits in Italy, Federico et al. [23] observed that in the 25–49 age group smoking prevalence was higher in the lower education group, both in men (OR of smoking in lower vs higher education: 1.71) and in women (OR: 1.12). In our study heavy smokers (defined as regular smokers of 11 or more cigarettes/day) were 26.5% of fathers and 3.2% of mothers: these figures were lower than those measured in a survey on smoking in Italian men and women in 2001, that reported an average 18.8 cigarettes per day in men and 12.2 in women [24]. However the difference may depend on a protective attitude of parents, who in spite of not stopping are likely to reduce the amount smoked, as was observed in studies on mortality by marital status and parenthood [25,26]. The same study also observed a lower prevalence in the North of Italy [24], corresponding to our results.

Compared to results on smoking exposure reported in recent international studies, our results shows similar prevalence of smokers among fathers and lower prevalence among mothers. The study carried out in France by Rudant et al. [27] investigated a European population with smoking habits not very different from Italian population: 5.6% of control mothers reported 10 or more cigarettes/day during pregnancy. The period considered for fathers (from birth to interview) is different from the one considered in our study, nevertheless it is of interest that 34.0% of fathers reported 10 or more cigarettes/day. In the study carried out in Canada by MacArthur et al. [28] prevalence of smoking (10 or more cigarettes per day) in pregnancy among controls was 21.6% for mothers, while and 40.6% of fathers of controls reported smoking before index child pregnancy. Lee et al. [29] in Korea reported that 80.4% of (hospital) controls’ fathers smoked in the year of child’s pregnancy. In Milne et al. [30] study, 12.3% of mothers of controls reported having smoked 15 or more cigarettes /day during pregnancy and 19.6% of fathers reported having smoked 15 or more cigarettes /day in the year of conception. The association of parental smoking and leukemia in the SETIL study was addressed by Mattioli et al. [31] for AnLL and by Farioli et al. [32] for ALL.

The SETIL study investigated occupational exposure to chemical substances and other agents using the expert assessment procedure. Results on solvents exposure were presented elsewhere [33]. Although such a procedure has been frequently used in occupational studies on adult cancer since the 80’s [34], its application in the investigation on parental exposure and childhood cancer has been limited so far. The different methodology in evaluating parental exposure and the different information provided in reports are serious limits in comparing the prevalence of exposure of controls across different studies and therefore only limited comparisons can be carried on. Solvent exposure was evaluated in different studies but only a few provided information useful for estimating parental prevalence of exposure: Bukley et al. [35] using self reported information evaluated that 61/178 controls had paternal solvent exposure and that in 23.6% (42/178) such exposure had lasted over 1000 days. Schuz et al. [36] estimated the prevalence of paternal and maternal exposure to solvents at any time before interview in 12.9% (382/2962) controls and in 5.0 (147/2962) of controls, respectively. Reid et al. [37] estimated the prevalence of paternal and maternal exposure to solvents at any time before child birth in 51% (382/748) controls and in 13% (114/854) of controls, respectively. Corresponding prevalence one year before birth was 25% (189/748) and 4% (31/854), respectively. In our study and considering controls, experts assigned exposure to solvents to 18.3% of fathers and 7% of mothers.

## Conclusions

Our results showed that the exposure of Italian children to cancer risk factors is not negligible, consistently with other countries of Western life style and industrial economy. Even if the association of common risk factors such as smoking with childhood leukemia is debated, they clearly represent risk factors for other diseases in childhood, such as asthma and acute respiratory illnesses. SETIL study results on prevalence of exposure indicate the need to take a stronger stance to reduce it, as it directly concern a large proportion of parents and indirectly affect their children.

Large size population based case control studies are a major resource for providing information on prevalence of exposure, as shown in this report. The Italian case–control study on childhood leukaemia, NHL and neuroblastoma (SETIL study) represents a useful source of data to estimate the prevalence of exposure of Italian children to a broad array of potential carcinogenic factors.

## Additional file

Additional file 1:
**Table S1.** Number of participating subjects by region and weight of each region. **Table S2.** Participation of mother and father at the interview, for a selection of the questionnaire sections. **Table S3.** SETIL study. Distribution by participation to ELF magnetic fields 48h measurements. **Table S4.** Occupational condition of controls’ parents from one year before conception until child’s diagnosis. **Table S5.** - Parental employment status of controls, by region.

## Abbreviations

ALL: Acute lymphoblastic leukaemia
AnLL: Acute lymphoblastic leukaemia
CI: Confidence interval
ELF-MF: Extremely low fields magnetic fields
LHA: Local health authority
NHL: Non Hodgkin lymphoma
RR: Relative risk
