# Supplementary material for: SETIL: Italian multicentric epidemiological case–control study on risk factors for childhood leukaemia, non hodgkin lymphoma and neuroblastoma: study population and prevalence of risk factors in Italy
Source: Ital J Pediatr. 2014 Dec 24;40:103. doi: 10.1186/s13052-014-0103-5 (PMC4310183; doi:10.1186/s13052-014-0103-5)
Supplement: Additional file 1: — Table S1. Number of participating subjects by region and weight of each region. Table S2. Participation of mother and father at the interview, for a selection of the questionnaire sections. Table S3. SETIL study. Distribution by participation to ELF magnetic fields 48h measurements. Table S4. Occupational condition of controls’ parents from one year before conception until child’s diagnosis. Table S5. - Parental employment status of controls, by region. [file 13052_2014_103_MOESM1_ESM.pdf]

SETIL: Italian Multicentric Epidemiological Case-Control Study on Risk factors for Childhood Leukaemia, Non Hodgkin Lymphoma and Neuroblastoma.

Study population and prevalence of risk factors in Italy

Additional tables

Table S1. SETIL. Number of participating subjects by region and weight of each region.

	Controls	Leukaemia	NHL	Neuroblastoma	Weight (£)	Notes
	n	n	n	n	%	
TOTAL PARTICIPANTS	1044	683	97	155	100	
PIEMONTE	88	61	9	23	8.9	*
LIGURIA	32	22	2	9	3.2	
LOMBARDIA	260	169	23	35	24.8	
VENETO	104	68	14	15	9.9	*
FRIULI V.G.	26	15	4	5	2.3	
EMILIA R.	59	35	2	5	5.1	**
TOSCANA	69	51	3	14	7.4	
UMBRIA	25	18	0	1	2.6	
MARCHE	41	20	4	7	2.9	
LAZIO	63	49	6	14	7.2	
CAMPANIA	49	33	10	9	4.8	*;**
PUGLIA	37	26	2	0	3.8	**
SICILIA (Eastern Provinces)	104	64	10	7	9.3	*
SICILIA (Palermo Province)	23	14	3	5	2	*; **
SARDEGNA	64	38	5	6	5.5	

£ proportion of leukaemia cases in the region over the study total, computed on participating cases only.

* Limited to part of the region.

** Study duration limited.

Table S2 SETIL study. Participation of mother and father at the interview, for a selection of the questionnaire sections. Only participating families are included.

			Controls		Leukaemia		NHL		Neuroblastoma	
			n	%	n	%	n	%	n	%
Sez 1 Overview and general information	Mother	yes, face interview	1029	98.6	672	98.4	97	100	153	98.7
		yes, telephone interview	2	0.2	0	0	0	0	0	0
		no	13	1.2	11	1.6	0	0	2	1.3
	Father	yes, face interview	621	59.5	472	69.1	61	62.9	107	69
		yes, telephone interview	33	3.2	9	1.3	1	1	4	2.6
		no	390	37.4	202	29.6	35	36.1	44	28.4
Sez 2 Pregnancy	Mother	yes, face interview	1031	98.8	673	98.5	96	99	154	99.4
		yes, telephone interview	2	0.2	0	0	0	0	0	0
		no	11	1.1	10	1.5	1	1	1	0.6
Sez 3 Mother's occupation	Mother	yes, face interview	1034	99	674	98.7	97	100	154	99.4
		yes, telephone interview	2	0.2	0	0	0	0	0	0
		no	8	0.8	9	1.3	0	0	1	0.6
Sez 4 Child's life	Mother	yes, face interview	1032	98.9	673	98.5	97	100	154	99.4
		yes, telephone interview	2	0.2	0	0	0	0	0	0
		no	11	1.1	10	1.5	0	0	1	0.6
Sez 5 Father's occupation	Father	yes, face interview	689	66	510	74.7	62	63.9	114	73.5
		yes, telephone interview	53	5.1	16	2.3	7	7.2	6	3.9
		no	302	28.9	157	23	28	28.9	35	22.6
Sez 6 Dwellings	Mother	yes, face interview	1026	98.3	667	97.7	97	100	154	99.4
		yes, telephone interview	2	0.2	0	0	0	0	0	0
		no	16	1.5	16	2.3	0	0	1	0.6
	Father	yes, face interview	609	58.3	463	67.8	58	59.8	106	68.4
		yes, telephone interview	21	2	4	0.6	2	2.1	3	1.9
		no	414	39.7	156	22.8	37	38.1	46	29.7

Table S3 SETIL study. Distribution by participation to ELF magnetic fields 48h measurements.

	Total	No interview	Interview and measurement	Interview, but no measurement	Measurements (out of total)	Measurements (out of interview participants)
	n	n	n	n	%	%
Controls	1475	431	904	140	61.1	86.6
Leukaemia	745	63	609	74	81.6	89.2
NHL	116	19	88	9	75.9	90.7
Neuroblastoma	207	52	135	20	64.6	87.1

Table s4 SETIL study. Occupational condition of controls' parents from one year before conception until child's diagnosis. Percentages are computed over the number of participant controls.

		Mother		Father	
Main groups of occupations	ILO code (1st digit) &	N *	%	N *	%
Total		1044		1044	
Professional, technical and related workers	0,1	180	17.3	182	17.6
Administrative and managerial workers	2	6	0.6	44	4.2
Clerical and related workers	3	222	21.3	130	12.6
Sales workers	4	70	6.7	90	8.7
Service workers	5	120	11.5	105	10.1
Agricultural, animal husbandry and forestry workers	6	16	1.5	49	4.7
Production and transport workers	7 - 9	110	10.6	443	42.8
Never employed		138	13.4	13	1.3
Missing		4		9	

& Based on the International Labour Office, Standard classification of occupation (ILO, Geneva Switzerland 1986) code at 1 digit levels.

TABLE s5 - SETIL Study. Parental employment status of controls (Ever worked vs. never), by region.

	MOTHER				FATHER			
	Never		Ever		Never		Ever	
REGION	N	%	N	%	N	%	N	%
Piemonte	5	5.7	83	94.3	3	3.4	85	96.6
Liguria	2	6.3	30	93.7	1	3.1	31	96.9
Lombardia	30	11.6	229	88.4	6	2.3	251	97.7
Veneto	2	1.9	102	98.1	0	0	104	100
Friuli V.G.	1	3.8	25	96.2	0	0	26	100
Emilia R.	1	1.7	58	98.3	2	3.5	56	96.5
Toscana	3	4.3	66	95.7	0	0	68	100
Umbria	1	4.0	24	96.0	0	0	25	100
Marche	1	2.4	40	97.6	0	0	41	100
Lazio	3	4.8	60	95.2	0	0	63	100
Campania	34	69.4	15	30.6	1	2	48	98.0
Puglia	9	25.7	26	74.3	0	0	34	100
Sicilia (East)	31	29.8	73	70.2	0	0	103	100
Sicilia (West)	9	39.1	14	60.9	0	0	23	100
Sardegna	6	9.4	58	90.6	0	0	64	100
TOTAL	138	13.3	903	86.7	13	1.3	1022	98.7
